# Dry Dosage Forms of Add-Value Bioactive Phenolic Compounds by Supercritical CO_2_-Assisted Spray-Drying

**DOI:** 10.3390/molecules27062001

**Published:** 2022-03-20

**Authors:** Clarinda Costa, Hugo Anselmo, Rita Ferro, Ana Sofia Matos, Teresa Casimiro, Ana Aguiar-Ricardo

**Affiliations:** 1 LAQV-REQUIMTE, Department of Chemistry, NOVA School of Science and Technology, Universidade NOVA de Lisboa, 2829-516 Costa da Caparica, Portugal; cid.costa@campus.fct.unl.pt (C.C.); h.anselmo@campus.fct.unl.pt (H.A.); rd.ferro@campus.fct.unl.pt (R.F.); teresa.casimiro@fct.unl.pt (T.C.); 2 Departamento de Engenharia Mecânica e Industrial, UNIDEMI, NOVA School of Science and Technology, Universidade NOVA de Lisboa, 2829-516 Costa da Caparica, Portugal; asvm@fct.unl.pt

**Keywords:** gallic acid, resveratrol, hydroxypropyl cellulose, dry powder, quality by design, encapsulation

## Abstract

Every year, grapevine pruning produces huge amounts of residue, 90% of which are from vine shoots. These are a rich source of natural antioxidants, mostly phenolic compounds, which, when properly extracted, can give rise to added-value products. However, their lack of solubility in aqueous media and high susceptibility to thermal and oxidative degradation highly limit their bioavailability. Encapsulation in suitable carriers may have a positive impact on their bioavailability and bioactivity. Previous data on vine-shoot extraction have identified gallic acid (GA) and resveratrol (RSV) as the main phenolic compounds. In this work, model dry powder formulations (DPFs) of GA and RSV using hydroxypropyl cellulose (HPC) as carriers were developed using Supercritical CO_2_-Assisted Spray Drying (SASD). A 3^2^ full factorial Design of Experiments investigated the solid and ethanol contents to ascertain process yield, particle size, span, and encapsulation efficiency. Amorphous powder yields above 60%, and encapsulation efficiencies up to 100% were achieved, representing excellent performances. SASD has proven to be an efficient encapsulation technique for these phenolic compounds, preserving their antioxidation potential after three months in storage with average EC50 values of 30.6 µg/mL for GA–DPFs and 149.4 µg/mL for RSV–DPF as assessed by the scavenging capacity of the DPPH radical.

## 1. Introduction

Viticulture is one of the most important economic activities worldwide, and it generates considerable pruning waste. Annually, about 1.5 tons per hectare of grape pruning waste (VPR) are generated, which translates into about 290 thousand tons per year in Portugal, much of which ends up being wasted or burned, causing harmful environmental effects [1]. The valorization of such lignocellulosic biomass in alignment with a circular bioeconomy may yield potential environmental impact reductions and increased profitability by giving rise to new added-value products [1,2]. According to the annual report of the International Organization of Vine and Wine (OIV) [3], global grape cultivation in 2018 covered about 7.5 million hectares, with Portugal being one of the ten countries with the most extensive vineyard coverage, with approximately 192 thousand hectares, with *Tinta Roriz* (TR), or *Vitis vinifera* L. [4,5] being the most common variety.

Vine shoots (or canes) [6], account for more than 90% of total viticultural waste [7]. They are lignocellulose biomasses enriched in natural bioactive compounds that may be converted into bio-based products by selective fractionation of the main components: lignin, cellulose, and hemicellulose. Several investigations into profitable applications for these residues have already been undertaken, such as the production of biochars, biofuels, cellulose for paper sheets, and lignin [8]. VPR composition studies have recently revealed a wealth of polyphenols, which, following the use of appropriate extraction techniques, are promising to produce bioactive compounds [9]. Polyphenols are an organic chemical class characterized by one or more aromatic rings with one or more hydroxyl groups. These secondary metabolites derived through the shikimic acid and phenylpropanoid pathways [10] are known for their beneficial effects and protective action on human health and as naturally occurring antioxidants broadly present in many parts of medicinal and woody plants, fruits and vegetables, including their peels, pulps and seeds [11]. 

Six main classes of phenolic compounds are found in grapevines, of which phenolic acids and stilbenes stand out [12]. These classes include the bioactive compounds under study: gallic acid (GA) and resveratrol (RSV), respectively. Various extraction techniques have been successfully applied to recover phenolic compounds from different vine shoot varieties, including the Portuguese varieties TR and *Touriga Nacional* (TN) [4,8,13,14,15,16,17,18]. Conventional extraction (CE) [19,20] is the most common. However, more sustainable, and efficient bioactive compounds extraction methods have been proposed, such as microwave-assisted extraction (MAE), supercritical-fluid extraction (SFE), subcritical-water extraction (SWE), and ultrasound-assisted extraction (UAE) [14,16,21]. It is important to note that both the extraction technique and experimental conditions [22] impact both the phenolic content as well as the phenolic distribution of the extracts. Recent VPR extraction studies on TN and TR Portuguese vines [4,8] have revealed that phenolic acids make up the largest portion of the extracts by SWE and MAE mainly due to the GA content, which varies between 26–78 %*w_GA_*/*w_dry sample_*. With SWE, the higher the extraction temperature, the higher the GA content extracted. VPR stilbenes extracts are mostly RSV, which appears in larger quantities only in the TR and TN extracts obtained using UAE, MAE and CE, with contents varying between 6–27 %*w*/*w*. The protective qualities of bioactive compounds, like polyphenolics, offer important promise for industrial applications. Encapsulation is one of the most auspicious and effective manners of protecting bioactivity and ensuring longer shelf life for these natural phenols [23]. A carrier, or coating material, inhibits crystallization in both dosage and in vivo forms, so that the bioactive compound is protected in the matrix until reaching the desired target [24]. Hydroxypropyl cellulose (HPC) and hydroxypropylmethyl cellulose (Hypromellose, or HPMC) are the most commonly used cellulose-based polymer carriers. HPC is a cellulose ether widely used in many food, pharmaceutical, and cosmetic formulations. It is Generally Recognized As Safe (GRAS) and is registered in the Inactive Ingredients Database approved by the FDA for both topical and oral formulations [25]. 

In some microencapsulation processes, HPC can be used as a thickening and coating agent to increase the bioavailability of heat-sensitive drugs in aqueous and acid systems at high temperatures [26], and it can act as a stabilizer and emulsifier in food and cosmetics [27]. Supercritical carbon dioxide-assisted processes have shown great potential to co-atomize and produce solid dosage forms of different bioactive compounds [28,29,30]. In particular, supercritical assisted atomization (SAA) and supercritical CO_2_-assisted spray drying (SASD) have both been used to produce dry powder formulations (DPFs) containing phenolic compounds [16,31]. Aliakbarian et al. [21] used supercritical assisted atomization (SAA) to encapsulate natural polyphenols extracted from the olive pomace, using maltodextrin (MD) as a carrier. To optimize atomization, different MD ratios were studied relative to the total extract solids (10 to 50% *w*/*w*), as were different drying temperatures (75 to 95 °C). Particles with diameters below 1 μm and with antiradical capacity were obtained. Di Capua et al. [31] assessed the feasibility and efficiency of SAA to encapsulate natural phenolic compounds in a propolis ethanolic extract, using hydroxypropyl-*β*-cyclodextrin (HP-*β*-CD) and polyvinylpyrrolidone (PVP) as carriers, with an encapsulation efficiency up to 100%. Moreover, the phenolic compounds maintained their bioactivity after SAA. SASD optimization is complex due to the large number of variables affecting the particles’ final properties. Design of experiments (DoE) is a well-established statistical method for improving optimization efficiency. Using an iterative process, a minimum number of experiments yield considerable knowledge about the system under study [32]. Studies of the application of DoE to CO_2_-assisted atomization optimization have been increasing [28,33,34,35]. This work attempted to develop dry powder dosage forms of GA and RSV by SASD using HPC as a carrier. A DoE approach investigated the influence of the process parameters (solid content and ethanol percentage) on the final powder properties (critical quality attributes). Physical, chemical, and aerodynamic characterizations were performed throughout to assess each formulation’s antioxidant activity.

## 2. Results and Discussion

### 2.1. SASD Conditions

Good efficiency and performance in atomization and particle formation depend on the selection of ideal process conditions. Such conditions are important for the formation of a homogeneous mixture in the static mixer, which is directly related to the phase equilibria of the supercritical carbon dioxide (scCO_2_) and the liquid solution [36]. The solubilization of the scCO_2_ in the liquid solution is promoted by a water/ethanol system since ethanol increases the water affinity in scCO_2_. A drying gas temperature near 100 °C is important to stimulate solvent evaporation from the droplets during atomization. Ethanol was also used to increase phenolic solubility in aqueous solutions. The parameters recorded during all the SASD experiments appear in Table S1 (Supplementary Materials).

Before the co-atomization of the carrier and bioactive compounds, some tests were performed to study the feasibility of processing HPC in the SASD apparatus. The ethanol content of 18.6% *v*/*v* in the feed solution, as well as the process operating conditions, were established according to previous studies on the same SASD apparatus and were set as optimal conditions [33,34]. In order to investigate the influence of the HPC content, solutions with 5.0, 7.5, and 10.0 %*w*/*v* of HPC were prepared in our first testing formulations. HPC concentrations of 10 %*w*/*v* showed high levels of viscosity, leading to nozzle clogging. So, solutions with 2.5, 5.0, and 7.5 %*w*/*v* of HPC were studied. It was possible to observe the formation of a fine, well-defined conical spray at the nozzle outlet. Microparticles with geometric sizes ranging from 18 to 20 µm were produced. 66% of the initial solids from the feed solution were recovered. 

**Figure 1:**
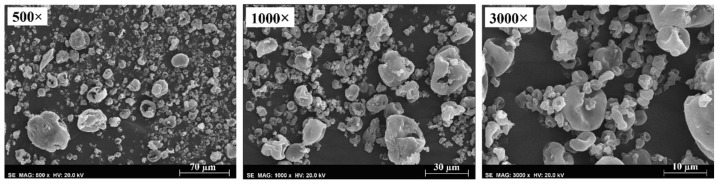
SEM images of HPC microparticles with 7.5 %*w*/*v* of HPC at 500×, 1000× and 3000× magnifications.

Some loss occurred at the internal walls of the precipitator chamber, possibly due to the typical static electricity following spray drying. This may have also been one of the factors affecting process yields. Physicochemical properties of the HPC microparticles appear in Table S2 (Supplementary Materials). Formulations with lower solid content were found to yield more homogeneous particle size distributions (lower span values). Morphologi G3 images also (Figure S2, Supplementary Materials) revealed good particle dispersion. At higher magnifications, some larger particles appeared to be aggregates of two or three particles. The DPF SEM images with the higher solid concentration in Figure 1 show that the microparticles appeared to be irregularly shaped yet slightly smooth, with the larger particles becoming more rounded.

Both unprocessed and SASD-processed HPC underwent solid-state characterization to identify if SASD had any direct impact on the polymer structure. Figure S3 (Supplementary Materials) shows that XRPD diffractograms revealed that both non-processed HPC and processed HPC exhibited two peaks at about 8° and 20°, indicating that this polymer has an amorphous domain. This aligns with data by Rahman et al. [37] that confirmed the same peaks for non-processed HPC. Differing HPC concentrations depicted the same peaks, although their intensity was shown to be slightly lower for the SASD HPC. In addition, the literature has also reported a few small crystalline peaks between 29–47° [38]. Rahman et al. [37] uncovered a small endothermic peak around 190–200 °C for the DSC thermogram of raw HPC. This probably results from the fusion of the small crystalline fraction of largely amorphous HPC or a liquid crystal isotropic transition [39,40]. Sarode et al. [39] predicted a low glass transition temperature, −20 to 0 °C, for this polymer, but the thermogram reported below did not detect one. Cellulose ethers typically show a glass transition of low intensity and the associated change in heat is too small to be detected by DSC [41]. ATR-FTIR measurements investigated DPF composition. Comparing the processed and unprocessed HPC powder, it was possible to conclude that HPC spectra were coincident (Figure S3, Supplementary Materials). All DPF maintained the same composition and chemical structure as the unprocessed ones following SASD.

### 2.2. Co-Atomization with Bioactive Compounds

After preliminary tests, the phenolic compounds were processed along with HPC in a bioactive/carrier ratio of 1:99 *w*/*w*. A DoE with the two parameters was applied. The DPFs were also subjected to a solid-state characterization as well as bioactivity and release tests in aqueous media.

#### 2.2.1. Statistical Analysis 

In view of process optimization, the goals were to maximize process yield and encapsulation efficiency, while minimizing particle size and span. Table 1 shows the results expressed as mean ± SD, considering the original set of nine experiments, plus one complete replication for RSV–DPF.

Table 2 summarizes the main ANOVA RSV–DPF results where the *p*-values of solid content and the ethanol percentages are associated with responses on process yield, encapsulation efficiency (*EE*), particle size (D_v,50_) and span. The linear and quadratic components are displayed whenever the effect was found to be significant (*p*-value < 0.05). Non-significant *p*-values appear as n.s. 

**Figure 2:**
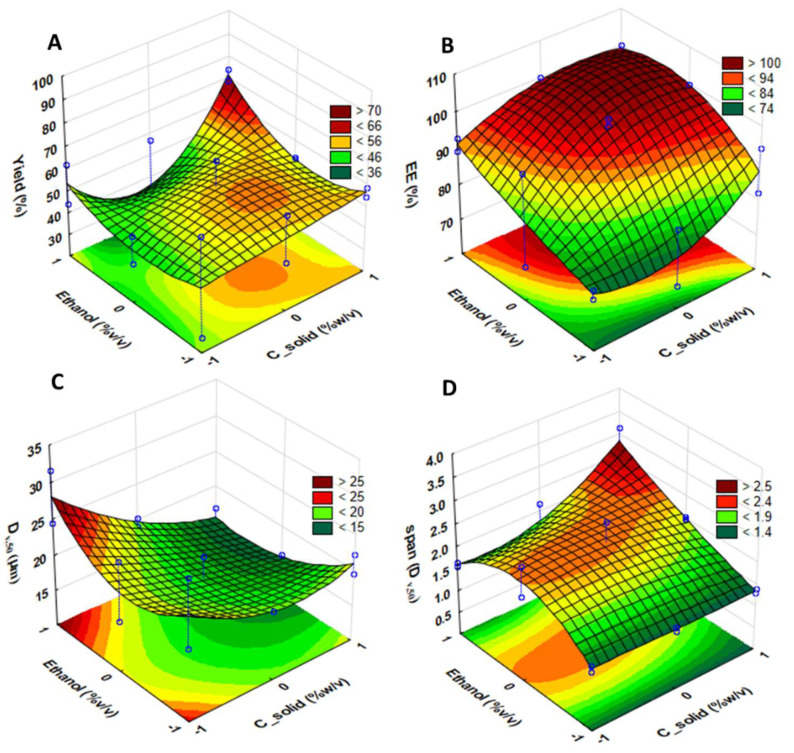
Fitted surface plots from ANOVA for each response (RSV–DPFs): (**A**) process yield; (**B**) encapsulation efficiency (*EE*); (**C**) particle size (D_v,50_); and (**D**) span.

In order to investigate the effect of each RSV–DPF (potential critical process parameters) on the critical quality attributes under study (process yield, encapsulation efficiency (*EE*), particle size (D_v,50_) and span), response surfaces for each variable were obtained using ANOVA (Figure 2). 

These surface plots reinforce the ANOVA significant effects (*p* < 0.05). Neither process yield C_solid content nor ethanol percentage were found to be statistically significant (*p* > 0.05), while they were so for the linear *EE. EE* increased with the rise of both C_solids and ethanol. Figure 2C shows that C_solids exhibited significant effects in the microparticle volumetric diameter in the linear component. Higher solid concentrations yielded smaller particles, regardless of ethanol percentage, contrary to what was expected. Particle width distribution was significantly affected by ethanol variable, while the C_solids showed a slight, but insignificant decrease in span values. Thus, ethanol was found to be significant in both the linear and quadratic components. Lower span values resulted when low and high levels were applied to the solution, regardless of solid content. A certain level of significance also appeared in the interaction of the linear components of both C_solids and ethanol, which is easily observed in the response surface. 

Table 3 shows the GA–DPF results as expressed as mean ± SD, considering the original set of nine experiments, plus one complete replication.

Table 4 summarizes the main ANOVA GA–DPF results, looking at the effects and *p*-values of solid content and ethanol percentage on the responses process yield, process yield, encapsulation efficiency (*EE*), particle size (D_v,50_), and span. 

**Figure 3:**
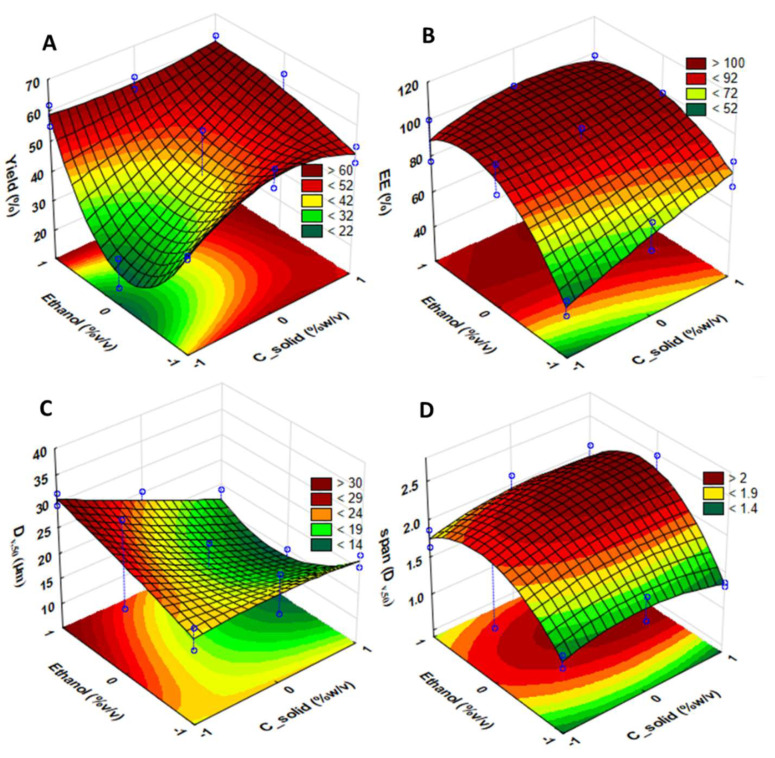
Fitted ANOVA surface plots for each GA–DPF response: (**A**) process yield; (**B**) encapsulation efficiency (*EE*); (**C**) particle size (D_v,50_); and (**D**) span.

Response surface plots for each variable in the HPC/GA formulation appear in Figure 3. 

For significance levels lower than 5% (*p* < 0.05), factor effects on the independent variables were identified. Ethanol affected the process yield to a high level of significance in the quadratic component. Intermediate ethanol percentages produced lower yields. C_solids displayed a significant effect on yield in the linear component. Also significant was the effect of ethanol (%*v*/*v*) on the linear component of C_solids in the quadratic component. Statistical significance was found for both variables in the GA *EE*. Increasing C_solids showed a linear increase of *EE*, while increasing ethanol from 20 %*v*/*v* to higher percentages was proven to have a greater impact on the linear component, although the quadratic component also affected *EE*. Figure 3C shows that, for the RSV–DPF, C_solids revealed a high significance level for the volumetric diameter of the GA–DPF microparticles in the linear component. Higher solution concentrations led to smaller particles. Finally, ethanol was significant in the quadratic component, as can be clearly seen in the corresponding response surface. Thus, the variance analysis validated our assumptions, namely, the normality of the residues, variance homogeneity, and residue independence.

#### 2.2.2. Solid-State Characterization

For RSV–DPFs, the mean particle volumetric diameter of 50% of the population (D_v,50_) ranged from 15 µm to 28 µm, for higher and lesser concentrations, respectively. The same trend obtained with the GA–DPFs, with mean volumetric particle diameters ranging from approximately 13 to 30 µm. Regarding the span, the closer the value was to 1, the narrower the range of particle size distribution and, therefore, the more homogeneous the population. Span values between 1.33–1.53 were recorded for both RSV and GA–DPF, and those closer to 1 resulted when the ethanol solution was the lowest and higher when ethanol percentages were over 45% (*v*/*v*). Overall, a narrow size distribution was observed for all DPFs. Scanning electron microscopy (SEM) evaluated the morphology and particle size of the bioactive compounds in their native form and following HPC processing. SEM images (Figure S4, Supplementary Materials) display the uniform rod crystal raw GA morphology with a broad particle size distribution, whereas raw RSV exhibited a geometric crystallized shape with a particle diameter of 0.1–30 µm.

**Figure 4:**
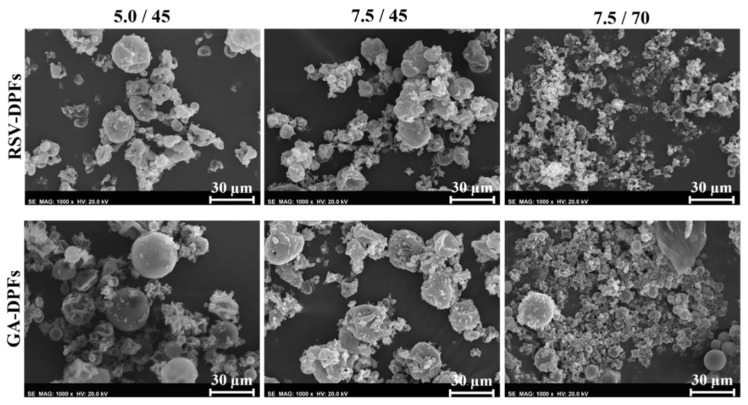
SEM images of both RSV–DPFs and GA–DPFs dry powder formulations with 5.0 and 7.5 %*w*/*v* of solids and 45 and 70 %*v*/*v* of Ethanol at 1000× magnification.

Figure 4 displays the morphology of the SASD-processed microparticles of RSV–DPFs and GA–DPFs (Figure 4) where broad particle size distribution and particle agglomerates for all0 three powders appear. These have a wrinkled surface analogous to HPC processed microparticles from the preliminary studies. This may be a result of the rapid solvent evaporation during decompressive atomization. Agglomerates may form as a result of HPC electrostatic and van der Waals forces due to high carbohydrate content. Smaller microparticles present rounded, irregular shapes while larger ones are spherical and smoother. For both 7.5 %*w*/*v* RSV–DPFs and GA–DPFs microparticles, increasing ethanol concentration from 45 to 70 %*v*/*v* decreases particle size distribution. No significant differences were observed for size distribution, only for solid concentration, as confirmed both by variance analysis and size and particle distribution.

The bioavailability of a model substance or drug of a DPF depends on the particle’s morphology and surface. Knowing the specific surface area of the co-atomized powders can help predict the dissolution rate, since the higher the specific surface area, the higher will be the dissolution rate [42]. The BET isotherms for both DPFs (Figure S5, Supplementary Materials) show a residual adsorption similar to type II isotherm. Hence, this isotherm is low and, any pores will be macropores. The vertical slope at the end of the isotherm may indicate macroporosity, but this must be confirmed by MIP (mercury intrusion porosimetry). However, the existence of residual nitrogen adsorption without defined hysteresis may also suggest some adsorption into the interparticular space rather than into pores. The gradual curvature (or “knee”) represented by point B indicates the monolayer coverage and the onset of multilayer adsorption. Point B estimates the amount of adsorbate needed to form a first monolayer as the subsequent multilayers appear at higher relative pressures [43].

As shown in Table 5, RSV and GA–DPFs have specific surface areas of 4.2 and 2.0 m^2^/g, respectively. GA–DPF values are in line with the specific surface area of the SASD HPC microparticles with different solid contents.

Karl Fisher results revealed residual moisture after co-atomization of RSV–DPFs and GA–DPFs in the range of 2.5–3.0% H_2_O/g_powder_, thus indicating good SASD drying efficiency. In a previously mentioned study, Aliakbarian et al. [21] encapsulated phenolic compounds into a natural extract with maltodextrin by SASD (SAA), yielding a moisture content from 3.1 to 0.6 %*w*/*w* [44].

**Figure 5:**
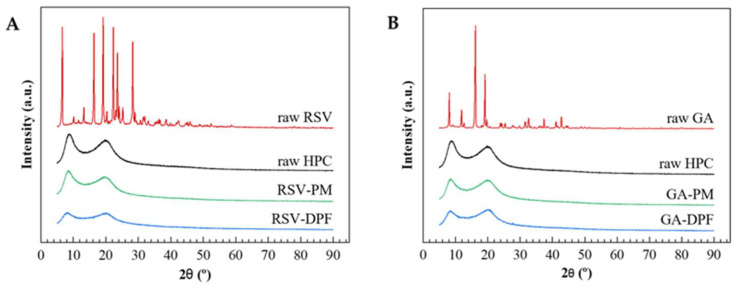
XRD diffractograms of (**A**) RSV–DPF and (**B**) GA–DPF; PM: physical mixture.

XRPD determined the physical state of raw phenolic compounds, as well as that of PM and DPF of RSV–DPF and GA–DPF. The diffractograms for both systems appear in Figure 5. Unprocessed RSV depicts many sharp peaks diffractions at approximately 2θ = 6°, 16°, 19°, 22°, 23.5° and 28°, whereas unprocessed GA depicts the main sharp peaks at approximately 2θ = 8°, 12°, 16° and 19°, confirming the crystalline state of both raw compounds. RSV and GA patterns agree with the literature [45,46].

At a high polymer/compound ratio (99:1 *w*/*w*), the PM resulting from both phenolics with HPC presents neither characteristic peaks of crystalline RSV nor GA, as expected due to the low amount of both phenolic compounds.

**Figure 6:**
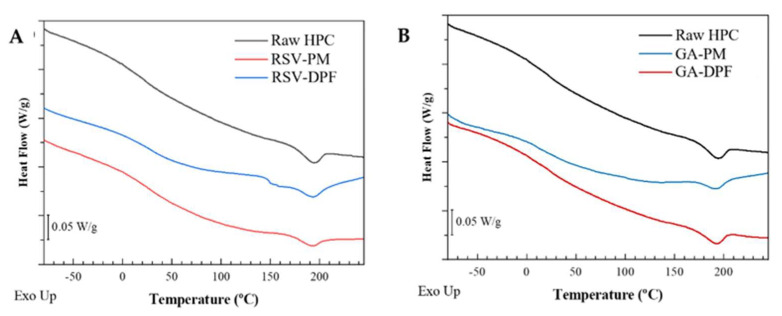
DSC thermograms (Run 2) for (**A**) RSV–DPF and (**B**) GA–DPF; PM: physical mixture.

DSC evaluated potential thermal behavior changes in the SASD powders and the polymer/compound mixtures. Both systems’ thermograms appear in Figure 6. Run 1 was not represented as it was equal to that previously reported, with the removal of water. The thermal profiles of the DPF and the physical mixture for both RSV–DPF and GA–DPF were identical to those of the raw HPC. All thermograms revealed a reproducible endothermic peak around 190–200 °C related to the melting point of the small crystalline domain of HPC, as previously discussed, suggesting that the melting temperature of the SASD-processed HPC was not affected by the addition of the bioactive compound.

The sharp melting peaks for the crystalline compounds (RSV and GA) reported in the literature usually appear in the range of 250–270 °C [47,48]. As the calibration limit of the DSC did not allow us to test the samples at higher temperatures, no potential changes in the thermal profiles of the PMs or DPFs could be detected. Theoretically, an endothermic melting event in the thermal profile close to the melting temperature of each phenolic compound would mean crystalline state encapsulation. The absence of such behavior would suggest that phenolic encapsulation takes place in an amorphous or molecularly dispersed state within the HPC [47], owing to the high content (~99.9 %*w*/*w*) of amorphous HPC in the physical mixture and in the dry powders. However, since the amount of the phenolic compounds is low compared to the carrier content, such peaks do not appear.

**Figure 7:**
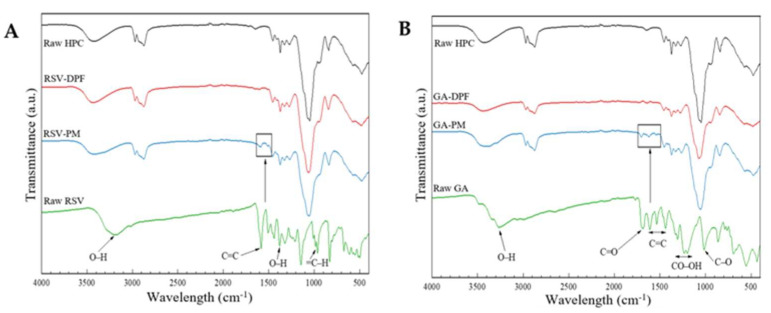
ATR-FTIR spectra for the (**A**) RSV–DPF and (**B**) GA–DPF; PM: physical mixture.

Figure 7 shows an overlapping of the ATR-FTIR spectra of the raw compounds, physical mixtures (PM) and DPFs, for both RSV–DPFs and GA–DPFs, respectively. The wavelength signals of both antioxidant compounds presented a broad peak around 3200 cm^−1^ associated with the phenolic hydroxyl bonds (O–H stretch). The RSV spectrum shows its characteristic absorption bands: at 1600 corresponding to the stretching of C=C bond of the aromatic ring; at 1580 cm^−1^ assigned to the stretching of the C=C olefinic bond; at 1380 cm^−1^ relative to the O–H bending; at 1143 cm^−1^ confirming the C–O stretching for phenolic compounds; at 965 cm^−1^ attesting to the alkene (=C–H bending) of the trans form of the RSV; at 828 cm^−1^ with respect to the =C–H vibration bands of arene conjugated to the olefinic group; and the deformation bands at 671–500 cm^−1^ corresponding to the =C–H of the olefinic group. The GA spectrum shows its characteristic absorption bands: at 1690 cm^−1^, corresponding to the stretching of C=O bonds of the carboxylic acid; at 1610, 1533, and 1440 cm^−1^, typical of the stretching vibrations of C=C bonds of the aromatic ring; in the 1300–1200 cm^−1^ range assigned to the CO–OH stretching and bending, indicating the combination of O−C–C asymmetric stretching and the OH bending; and at 1020 cm^−1^, related to the C–O stretching. These results were in agreement with the literature for both phenolic compounds [49,50,51].

A close look at the low-intensity bands at 1589 and 1500 cm^−1^ for the physical mixture of RSV and HPC at 1703, 1618 and 1540 cm^−1^ and for the physical mixture of GA and HPC reveals evidence of slightly more intense peaks relative to the raw HPC spectrum in that region. As they are more characteristic of the raw RSV and GA, these peaks may show up in the sample, since these are in very small proportion (1 %*w*/*w* sample). The DPF samples present spectra like unprocessed and processed HPC (in the preliminary studies), suggesting that the compounds are probably not detected at the particle surface.

#### 2.2.3. Antioxidant Activity

**Figure 8:**
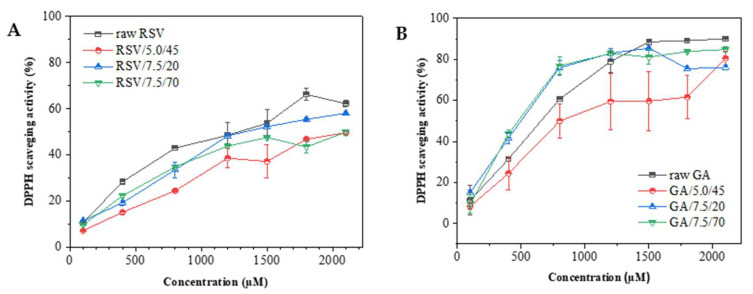
Antioxidant activity of raw materials and SASD-processed formulations with (**A**) RSV and (**B**) GA.

Antioxidant activity tests assessed whether the RSV or GA co-atomized with HPC by SASD maintained their ability to eliminate DPPH free radicals, compared to both bioactive compounds in their pure forms. Three SASD powders considered the best in encapsulation efficiency were studied. Thirty minutes following the blend of the DPPH• and compounds, a certain degree of color change from deep violet to a yellowish color was expected to indicate the scavenging potential of the antioxidants. In other words, the free radical DPPH• in the reaction solution is reduced to its non-radical form (DPPH•-H). Figure 8 shows the percentage of DPPH radical scavenging plotted as a function of RSV and GA concentration.

Figure 8A shows that all the processed DPFs have a slightly lower antioxidant activity than the raw forms, among which those with higher solid content, namely RSV/7.5/20 and RSV/7.5/70, exhibit greater antioxidant activity. This indicates that higher RSV concentrations in the initial SASD feed formulation increase the final DPF antioxidant capacity. The curve tends to stabilize for all samples as concentrations reach 2000 µM, meaning that the RSV antioxidant power is depleted as it acts as a limiting reagent. Figure 8B shows that the two GA–DPFs with higher solid content (GA/7.5/20 and GA/7.5/70) presented greater antioxidation up to about 1300 µM. Those with lesser solid content (GA/5.0/45) consistently demonstrated lower antioxidation as a result of lower phenolic loading in the formulation. The curve fluctuates, however, more due to the greater associated error and the last point suggests an activity increase similar to other formulations. After 1250 µM, all sample curves begin to stabilize, which may correspond to the near-total DPPH radical conversion.

The effective concentration (EC_50_) represents the minimum antioxidant concentration needed to enable the DPPH free radical inhibition by 50%. As per Alexander et al. [52], some precautions were taken. Table 6 summarizes the EC_50_ values for RSV and GA. It is worthy of note that the samples were stored for three months before antioxidant activity was assessed.

Lower EC_50_ values indicate higher free radical scavenging by the antioxidant. All DPFs showed higher EC_50_ value than the raw form, except for that with 7.5 %*w*/*v* of solids and 70 %*v*/*v* of ethanol (RSV/7.5/70), which presented a EC_50_ value of 110.36 µg/mL, i.e., greater antioxidation than the pure compound. The fact previously displayed in SEM images that formulations with more solids and higher ethanol percentages yield smaller particle sizes, may have an impact on DPF antioxidation. Smaller particles increase the surface area in contact with the reaction medium [45]. The formulation with the lowest solid content (RSV/5.0/45) presented the highest EC_50_ value, thus corresponding to the powder with the lowest antioxidant potential.

The EC_50_ values of DPPH free radical scavenging by GA ranged from 27 and 37 µg/mL. As with RSV, pure GA EC_50_ values revealed less antioxidation than the two formulations with higher solids. In fact, a decrease of approximately 25% of the EC_50_ was observed for those powders processed with 70 %*v*/*v* of ethanol. The maximum temperature these bioactive compounds can be subjected to during SASD is one of the main factors affecting antioxidation. However, as seen by comparing both DPFs and pure compound antioxidation, temperatures around 100 °C in the SM and 60 °C in the cyclone at higher ethanol content did not affect the activity of RSV–DPF but did influence the GA–DPF activity. This is promising, suggesting that the carrier provides efficient protection, and that the RSV remains active (*trans-*). Any bioactivity losses must not be associated with atomization since the particles are only subjected to these temperatures for short periods. Rather the cause would appear to be the resting time of the final DPF in the collection flask until room temperature is reached at the end of each SASD assay.

Comparing both phenolic compounds reveals that GA powders are the most effective scavengers eliminating 50% of the initial DPPH radical. That is, at lower GA concentrations, greater RSV antioxidation takes place. This might be attributed to the higher number of active GA hydroxyl groups in the structure of GA than those of RSV. The hydroxyl groups can establish hydrogen bonds with the DPPH free radical, stabilizing it [53]. Our supercritical fluid techniques powder EC_50_ values were relatively higher than those in the literature. Dal Magro et al. [45] tested antioxidation of RSV/PHBV coprecipitated particles using SESD. These powders’ EC_50_ values ranged from 10–29 µg/mL, higher than the antioxidant activity observed for the raw RSV (28 µg/mL). Aguiar et al. [54] also studied the antioxidation of micronized RSV by the SEDS (without carriers) and obtained EC_50_ values in the range of 26–37 µg/mL, as compared with 28 µg/mL of the raw RSV. The DPPH^•^ elimination activity plotting data is given as a function of concentration in Tables S5 and S6 (Supplementary Material).

#### 2.2.4. In Vitro Aerodynamic Performance

**Figure 9:**
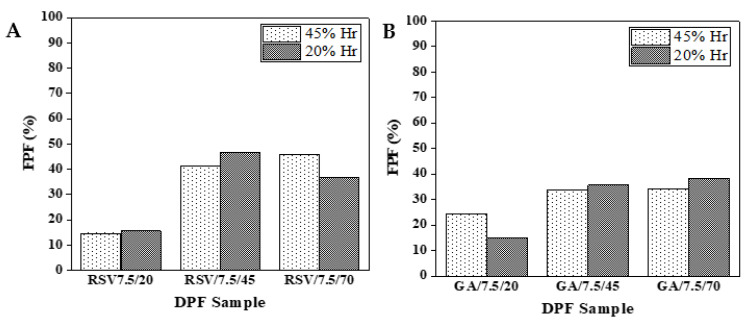
Fine particle fraction (FPF) for 7.5 %*w*/*v* DPFs stored at different %RH, varying ethanol volume from 20 to 70 %*v*/*v*: (**A**) RSV–DPF; (**B**) GA–DPF.

An Andersen Cascade Impactor (ACI) evaluated the aerodynamic behavior of the DPF stored at different humidity (20 and 45% RH) DPFs with equal solid contents (7.5 %*w*/*v*) and those processed with varying SASD ethanol percentages. Figure 9 displays that the respirable fraction (FPF) most likely to deposit in the deep lung does not exceed 50 and 40% for RSV–DPF and GA–DPF microparticles, respectively. This may be due to the presence of many aggregates, which retain a significant amount of powder in the upper airways. The highest FPF values were for those DPFs with an ethanol percentage atomized of at least 45 %*v*/*v*, which may suggest that the powder is looser. Moreover, no RH influence was found for powder aerodynamic performance. FPF values were below those for powder formulations produced by this SASD apparatus (58–86%) [28,33,34]. In these formulations, other carriers were used and demonstrated better aerodynamic lung delivery.

**Figure 10:**
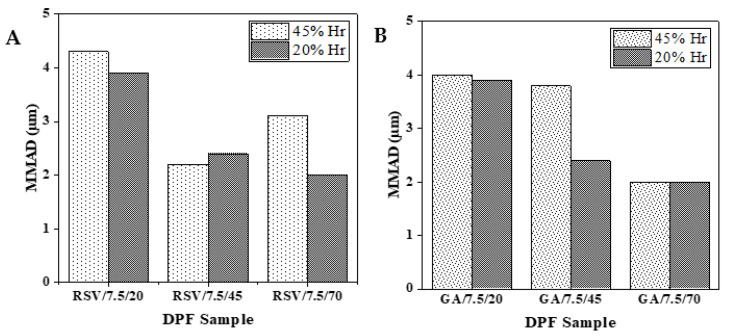
Mass median aerodynamic diameter (MMAD) for 7.5 %*w*/*v* DPFs stored at different %RH, varying ethanol from 20 to 70 %*v*/*v*: (**A**) RSV–DPFs (**B**) GA–DPFs.

Particle MMADs in Figure 10 indicate aerodynamic diameters within a range (0.5–5 µm) that should allow deep lung delivery. Any larger particles will be trapped in the upper airways, while smaller ones will be exhaled and fail to deposit [55]. Higher MMAD levels results for RSV and GA–DPFs prepared with lower ethanol percentages, due to the formation of a greater numbers of aggregates, as mentioned previously.

#### 2.2.5. In Vitro Phenolic Release

**Figure 11:**
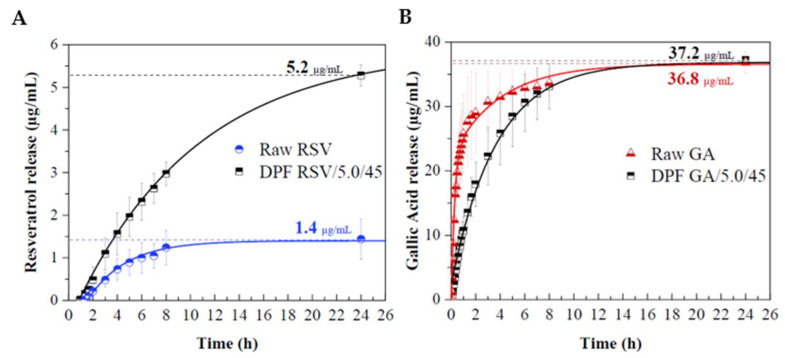
In vitro phenolic release profiles in pH 5.5 PBS at 32 °C for 24 h of (**A**) raw RSV and DPF RSV/5.0/45 samples and (**B**) raw GA and DPF GA/5.0/45 samples, expressed in µg of phenolic per mL of solution.

Another key data point capable of confirming successful co-atomization and phenolic compound bioavailability is the release profile. We evaluated the in vitro phenolic release from the DPFs in an aqueous medium at pH 5.5 and 32.0 ± 0.1 °C. These sink conditions were chosen to mimic in vitro skin absorption, as healthy human skin must be maintained at a temperature of 32 ± 1 °C with a pH between 4.5–5.5 [56]. Two DPFs received identical preparations (5.0 %*w*/*v* solids, 45 %*v*/*v* ethanol). The previously determined phenolic concentration of HPLC for the *EE* was 1.017 and 880 µg per 100mg sample, respectively for RSV–DPFs and GA–DPFs. Figure 11 shows the percentage of phenolic compound released over 24 h, normalized by the maximum amount of RSV and GA released. As is obvious, the maximum mass released for both phenolics is achieved more quickly in their free forms than in the encapsulated ones. RSV release only becomes detectable after 1 h. The R SV-DPF curve represents a controlled release of RSV over time, reaching 50% of the total mass released after 8 h, while the free RSV reaches 50% of its maximum in the first 4 h. Maximum RSV mass only occurred after 48 h. GA was detected quickly in the first few minutes. After 25 min, 40% of its maximum released mass of free GA was reached while encapsulated GA released 6% of its maximum in the same period.

**Figure 12:**
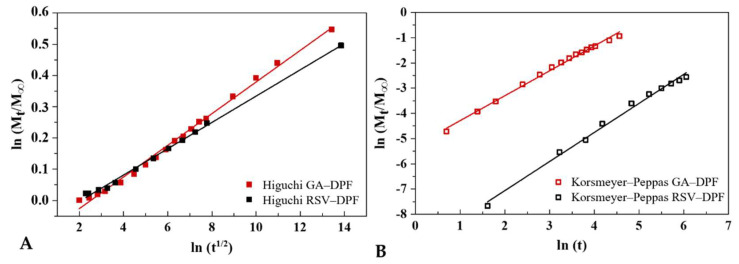
RSV and GA release profile from RSV–DPF and GA–DPF microparticles by (**A**) Higuchi and (**B**) Korsmeyer–Peppas mathematical models.

Figure 11A reveals that the raw RSV release rate seems to gradually increase in the first 8 h, after which it begins to stabilize up to a concentration of 1.4 ± 0.4 µg/mL. The enhanced dissolution rate of encapsulated RSV–DPF compared to the free RSV may be attributable to the increased specific surface area (from 2.19 m^2^/g of raw RSV to 4.20 m^2^/g of RSV–DPF) [57]. The amorphous nature of encapsulated RSV lends it higher solubility than crystalline RSV and, thus, quicker dissolution [58]. Clearly, the release rate falls over time for both samples. The GA maximum is reached at about the same time for both free (raw) and encapsulated GA. A slightly more controlled release rate is observed for encapsulated GA (Figure 12B). GA release from the GA–DPF starts to stabilize after 8 h, decreasing with time. An analogous result was reported by Robert et al. [59] for the release of GA from GA-starch and GA-inulin microparticles in water at 25 °C. They named this rapid release rate the “burst effect”, due to the initial release of the superficial GA.

RSV release was well below that initially estimated in the DPF. Problems dissolving the compound may also be associated with the fact that the membrane is floating on the liquid surface, with little space to move. Although RSV solubility is difficult to predict at different pH’s, it varies between 64 µg/mL at pH 1.2 and 61µg/mL at pH 6.8 [60] in acidic media. Being a hydrophilic polymer soluble at all pH conditions [61], HPC may also have facilitated RSV dissolution from DPF. GA release, both on the free and DPF forms, almost reached the initial amount placed in the particle. Zhang et al. [57] studied resveratrol nanoparticle dissolution (by antisolvent precipitation and spray-drying) in a pH 6.8 solution at 37 °C, with full dissolution of RSV/HPMC nanoparticles at 45 min (30 µg/mL), whereas raw RSV only completely dissolved after 120 min (22.5 µg/mL). Dimer et al. [62] reported a release of over 80% of raw RSV in aqueous medium at 37 °C after 30 min, whereas RSV/poly(ε-caprolactone) spray-dried microparticles had a more controlled release, reaching 80% after 8 h. Ha et al. [47] reported a maximum concentration of *trans-* RSV/HPMC nanoparticles (by SAS) released in distilled water at 37 °C of 150 µg/mL after 24 h. Cardoso et al. [63] released RSV from arabic gum microparticles produced (by spray-drying) in coconut oil at 37 °C, reaching a maximum concentration after 15 min. The release profiles are modelled using the Higuchi and Korsmeyer–Peppas mathematical models (Figure 12).

Both models were able to correlate the experimental release curves (Table 7). The Higuchi model shows that the release mechanism is described by Fickian diffusion [64]. As the diffusional release exponent (*n*) values obtained with Korsmeyer–Peppas model are well above 0.85 for both RSV–DPF and GA–DPF, the release of the phenolic compounds was governed by a Case-II transport. Thus, the molecule release is explained by the relaxation of the chains of the polymeric matrix when in contact with the aqueous medium. Due to high concentration gradients at the polymer/water interface, the cellulosic structure of the HPC swells, and the water is imbibed into the matrix [65]. The release rate constant (*k*) values were higher for the GA–DPF, indicating a faster release rate for the GA than that of RSV. This corroborates the release rates results observed in the in vitro profiles.

#### 2.2.6. Quantification Tests

**Figure 13:**
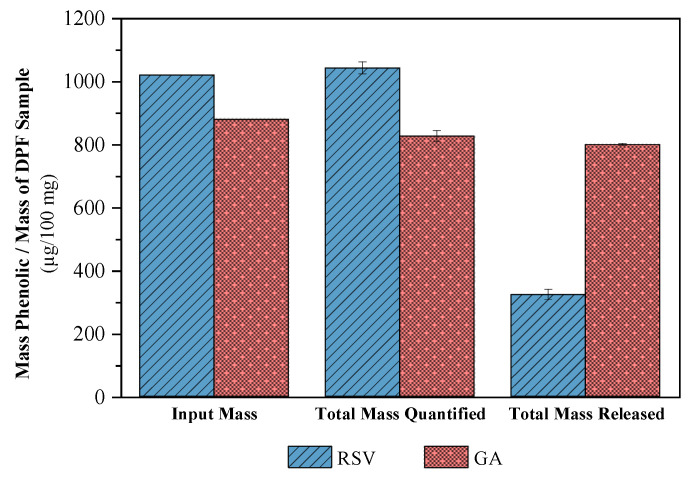
Input, total quantified (in ethanol), and total released (in pH 5.5 PBS) masses of RSV and GA expressed in µg of phenolic per 100 mg of total DPF sample.

A repeat quantification test was carried out on the samples in the previous release assays. Phenolic content dissolved in ethanol was measured to prove the results obtained from HPLC for *EE*. Secondly, the DPFs predicted to appear in the pH 5.5 PBS solution were placed to dissolve directly into the medium. The total amount of RSV and GA dispersed in the ethanolic medium (Figure 13), (1043 ± 19 and 826 ± 18 µg/100 mg DPF, respectively) was practically equivalent to that of RSV and GA in the *EE* tests (1020 and 880 µg/100 mg DPF, respectively). Thus, the encapsulation efficiency of each DPF used in these tests is corroborated. The maximum concentration in the previous release profiles is confirmed, with relatively coincident values for both PDFs. However, in terms of the total amount of RSV in the solution, only about 30% of the initial input was dissolved. For GA, the release was about 97%, almost total. HPLC chromatograms for the HPC samples (without any of the phenolic compounds) failed to detect any peak polymer area at the compound detection wavelengths. This confirms that the polymer did not mask the results of the RSV–DPFs and GA–DPFs samples in HPLC.

## 3. Materials and Methods

### 3.1. Material

Hydroxypropyl cellulose (HPC, Mw ~18,000), gallic acid monohydrate (GA, ≥98.0% purity, Mw = 188.13 g/mol) and 2.,2-Diphenyl-1-picrylhydrazyl (DPPH, Mw = 394.92 g/mol) were purchased from Sigma-Aldrich (Burlington, MA, USA) and Resveratrol (RSV > 99.0% purity) was sourced from Tokyo Chemical Industry (Tokyo, Japan). Deionized water was prepared by reverse osmosis (Milli-Q, Millipore). Ethanol absolute anhydrous (ethanol, 99.8% purity) was purchased from Pan-Reac ITW Reagents (Barcelona, Spain). All compounds were used as received without further purification. Industrial carbon dioxide (purity ≥ 99.93%) from Air Liquid (Paris, France) was used. Phosphate Buffer Solution (PBS) pH 5.5 was prepared according to European Pharmacopeia [66].

### 3.2. Formulation and SASD Apparatus

A solution was prepared with each phenolic compound (GA and RSV), separately to create a model system with a composition of a natural phenolic extract. 1 %*w*/*w* of phenolic compound was present in each formulation, with the rest comprised of biopolymer hydroxypropyl cellulose (HPC) as carrier. While the HPC solution was prepared in deionized water and continuously stirred for several hours before processing, both GA and RSV ethanol solutions were prepared shortly before the process and mixed with the HPC aqueous solution. The solution was then filtered into an amber flask to protect the bioactive compounds from light while it was fed into the process. The experiments took place in a SASD laboratory-scale apparatus described elsewhere [34]. In this process, two independent streams were fed into the apparatus: (a) a homogeneous liquid feed containing both bioactive compound and the carrier was pumped through a high-pressure liquid pump (Smartline Pump 1000, Knauer, Berlin, Germany); (b) liquefied CO_2_ from a cylinder (TP) was first cooled in a cryogenic bath and then subjected to a high-pressure liquid pump (HPLC pump K-501, Knauer). Liquefied CO_2_ was heated in an oil bath and then sent to the static mixer (SM) to enable solubilization and homogenization with the solution, creating near-equilibrium conditions before atomization. Pre-atomization pressure at the SM was controlled by a Setra (Boxborough, MA, USA) pressure transducer (0.1 psig stability). A Shinko (Osaka, Japan) temperature controller guaranteed SM temperature with heating tapes. The mixture was then depressurized through a nozzle and sprayed into an aluminum precipitator (with a polycarbonate window frame) at near-atmospheric pressure. Simultaneously, inlet heated compressed air flow at 30 kg/h evaporated the liquid solvent from droplets to form particles. The particles exited from the bottom of the precipitator to a high-efficiency cyclone for separation from the gas stream and collection in a glass flask. The powder was stored in amber flasks inside a desiccator.

### 3.3. Design of Experiments

A 3^2^ full factorial design was applied to study two formulation variables in SASD optimization for producing HPC-based DPFs with GA or RSV: 8 points plus 1 central point (Figure S1, Supplementary Material). The effect of the solid contents (C_solids, %*w*/*v*—Factor A) and ethanol content (Ethanol %*v*/*v*—Factor B) in the liquid solution were assessed with respect to the process yield, particle size, span, and encapsulation efficiency of the bioactive compound in the polymeric matrix. Solid contents ranged from 2.5–7.5 %*w*/*v*, whereas the ethanol composition varied between 20–75 %*v*/*v.* A total of 18 replications were run for each factor combination and for each compound. Any potential correlations of the dependent and independent variables as well as their significance levels for the various experiments were analyzed by Statistica V12 (StatSoft, Inc., Hamburg, Germany) software.

A 3^2^ full factorial DoE was performed to test all combinations of factor levels, two by two, resulting in nine different combinations (Tables S3 and S4, Supplementary Materials). The critical process parameters (CPPs), initial solid content and ethanol percentages were assessed in relation to the critical quality attributes (CQAs) selected, the process yield, particle size, span and encapsulation efficiency. ANOVA identified, for a significance level less than 5% (*p* < 0.05), the significant effects of the factors for each variable. The three-level factors were studied both linearly and quadratically. A significant effect on the linear component yielded the best response to the factor at the high (+1) and low (−1) levels. A significant effect in the quadratic component implied that the best response was at the intermediate level (0); a lack of significance was also found between the intermediate (0) and extreme levels (−) or (+). Thus, the best level response was one of the extreme ones.

### 3.4. Process Yield and Encapsulation Efficiency

The process yield (Equation (1)) was determined by the ratio between the power mass remaining at the end of the process, *m_p_*, and the solid content uploaded into the initial feed solution, *m_s_*.

(1) $$\eta \;(\%)=\frac{m_{p}}{m_{s}}\;\times \;100$$

High-Performance Liquid Chromatography (HPLC) measured the quantities of the encapsulated phenolic compound in each RSV–DPF and GA–DPF, as per Ahmad et al. [67] with some modifications. A Thermo Kromasil Keystone C18, 5 µm, 4.6 mm × 250 mm column and a UV-Vis detector assessed the samples. The mobile phase (eluent) consisted of a mixture of 0.5 %*v*/*v* acetic acid in methanol: water (50:50 *v*/*v*), pumped at a flow rate of 1 mL/min and at 30 °C. The solution resulted from dissolving 1 mg of DPF in 1 mL of eluent. 20 µL was injected into the HPLC column. RSV and GA were identified by comparing the retention time and the detected peaks by UV-Vis spectra with those obtained for standards. Retention times of approximately 6 and 2.7 min were observed for RSV and GA, respectively. The RSV–DPF solutions were read at a wavelength of 304 nm whereas the GA–DPF solutions were read at 280 nm. Then, to quantify the RSV or GA in the DPFs, the peak area given in the HPLC chromatogram was replaced in the linear regression obtained by the standard point of phenolic concentration versus peak area. Encapsulation efficiency (*EE*) was determined by the ratio of the encapsulated mass detected by HPLC and the phenolic mass input (Equation (2)).

(2) $$EE\;(\%)=\frac{\mathrm{Mass}\;\mathrm{of}\;\mathrm{compound}\;\mathrm{encapsulated}}{\mathrm{Input}\;\mathrm{mass}\;\mathrm{of}\;\mathrm{compound}\;}\;\times \;100$$

### 3.5. Microparticle Characterization

#### 3.5.1. Particle Size and Particle Size Distribution

Particle size (PS) and particle size distribution (PSD) were measured in a Morphologi G3 device (Malvern Instruments, UK). To ensure good feasibility, at least 30,000 particles were photographed and statistically analyzed according to different geometrical factors, namely, particle volume diameter (D_v_) and number (D_n_) corresponding to 10, 50 and 90% of the total population. Particle width distribution (span) was also determined using D_v,10_, D_v,50_ and D_v,90_, as represented in Equation (3).

(3) $$\mathrm{Span}=\frac{D_{v90}-D_{v10}\;}{D_{v50}}$$

#### 3.5.2. Particle Morphology

The shape and surface morphology of the atomized DPFs in the SASD were observed by Scanning Electron Microscopy (SEM). The samples were glued to adhesive carbon tapes and excess powder was removed using compressed air. The samples were then analyzed by a Hitachi S2400 (Tokyo, Japan) with an accelerating voltage set to 15 kV and at magnifications of 500, 1000 and 3000×.

#### 3.5.3. X-ray Powder Diffraction (XRPD)

The solid characterization of the native compounds, physical mixture (PM) and DPFs was carried out using X-Ray Powder Diffraction (XRPD). The PMs were prepared in the same HPC/compound ratio as the SASD formulation by gently mixing with a ceramic mortar and pestle. The measurements were performed in a RIGAKU X-ray diffractometer (model Miniflex II) with automatic data acquisition (Peak search for Windows v. 6.0 Rigaku) using CuKα radiation (λ = 0.15406 nm or 1.54 Å) and working at 30 kV/15 mA. Diffraction patterns were collected in the range 2θ = 5–90° with a 0.02° step size and an acquisition time of 1°/min.

#### 3.5.4. Specific Surface Area

The specific surface areas (expressed in m^2^/g) of the native compounds and DPFs were measured by adsorption porosimetry with N_2_ at 77 K, as per Badawy et al. [68]. The powder was first loaded into a sample cell and degassed by N_2_ flow for at least 2 h under vacuum at 60 °C. The data was collected by a Surface Area Analyzer ASAP Model 2010 (Micromeritics Instrument Corporation, Norcross, GA, USA) using the Brunauer, Emmett, and Teller (BET) equation.

#### 3.5.5. Attenuated Total Reflection-Fourier Transform Infra-Red (ATR-FTIR)

ATR-FTIR investigated the interactions between the carrier and the bioactive compound. Samples of raw phenolic compounds, two DPFs, with each compound and their physical mixtures (PM) were analyzed. The mixture preparations used the method described above. A Spectrum Two FTIR spectrometer (PerkinElmer, Inc., Waltham, MA, USA) carried out the analysis. A proper amount of each sample was placed in a crystal diamond plate, completely covering the prism surface. Each spectrum was scanned 16 times from 400 to 4000 cm^−1^.

#### 3.5.6. Differential Scanning Calorimetry

DPF and PM thermal behavior was assessed using differential scanning calorimetry equipment (DSC Q2000, TA Instruments, New Castle, DE, USA) with a refrigerated cooling system. The physical mixtures were prepared by the same method as above. Approximately 5 mg of sample were accurately weighed into a hermetically-crimped pinhole aluminum pan (Tzero Hermetic Lid, TA) and heated under a continuous dry nitrogen purge (50 mL/min) for two runs. The first heating ramp (Run 1) started at –90 °C and increased to 250 °C, at a heating rate of 10 °C/min, to eliminate any sample water. A cooling ramp then lowered the temperature to −90 °C at a rate of 10 °C/min, before starting the second heating ramp (Run 2) to 250 °C again, at the previous rate. Here, either the glass transition or the melting point can be identified by a sigmoidal difference in the thermal baseline or by an endothermic peak, respectively. DSC data and thermograms were extracted by TA Instruments software.

#### 3.5.7. Moisture Content

Residual moisture in the atomized DPFs was determined using the Karl Fisher (KF) titration method (756/831 KF Coulometer, Metrohm Ion Analysis, Ltd., Herisau, Switzerland). About 30 mg of DPF sample were accurately weighted and dissolved in 600 µL of anhydrous ethanol. The sample was continuously stirred until fully diluted. An aliquot of 0.3 mL was removed from the solution and weighed before and after titration, and the comparison of the two weights yielded the moisture content. The experiment was performed in triplicate.

### 3.6. Antioxidant Activity

DPF antioxidant activity was assessed by the scavenging capacity of the DPPH radical, as per Medina-Torres et al. [49]. Similar tests for each pure phenolic compound (RSV and GA) served as a comparison to the DPF antioxidation. The samples were prepared in triplicate for each concentration and kept in the dark (in an amber vial) for 30 min at room temperature. Absorbance of 517 nm was measured in a EvolutionTM 201 UV-Vis spectrophotometer with Thermo Insight software (Thermo Fisher Scientific, Waltham, MA, USA) by adding an aliquot of sample to a 1 mL cuvette. DPPH free radical scavenging was determined using Equation (4) and plotted against concentration. EC50, defined as the minimum effective concentration of antioxidant necessary to scavenge 50% of the initial DPPH free radical, was determined using the Alexander et al. method [52].

(4) $$\%\;\mathrm{DPPH}\;\mathrm{radical}\;\mathrm{scavenging}\;\mathrm{activity}=\frac{A_{\mathrm{DPPH}}•-A_{\mathrm{sample}}}{A_{\mathrm{DPPH}}•}\;\times \;100$$

### 3.7. In Vitro Aerodynamic Performance

DPF aerodynamic performance was evaluated gravimetrically using an 8-stage aluminum Andersen Cascade Impactor (ACI) apparatus (Copley), in accordance with the European Pharmacopeia [69]. The DPFs were first stored for two weeks at differing relative humidity (20 and 45% RH) to evaluate their aerodynamic performance. For each assay, approximately 30 mg of powder was hand-filled in hypromellose (HPMC) n° 3 capsules (Aerovaus) which were then placed in a handheld breath-activated inhaler (Aerolizer Plastique 60 LPM–Model 7 dry powder inhaler (DPI)) attached to the ACI inlet fixed to a horizontal testing stand. A high-capacity pump model HCP5 (Copley) maintained the flow rate through the sampling apparatus to simulate inhalation and the tests were performed in triplicate. The inhaler and all the glass microfiber filters (MFV1 80 mm, Filter Lab, Barcelona, Spain) were weighed on an analytical balance prior to the test. The filters were placed in all plate stages before the assay. After releasing the three capsules, the total powder mass was determined for each stage filter. The capsule and inhaler were weighed to determine the amount of residual powder. The fine particle fraction (FPF) was determined by the interpolation of the percentage of the particles collected in each ACI experiment containing an aerodynamic diameter less than 5 μm. The mass median aerodynamic diameter (MMAD) was determined as the particle diameter corresponding to 50% of the cumulative distribution. The geometric standard deviation (*GSD*) was determined by the following Equation (5):

(5) $$GSD=\sqrt{\frac{d_{84}}{d_{16}}},$$

where *d*_84_ and *d*_16_ are the diameters corresponding to 84 and 16% of the cumulative distribution, respectively.

### 3.8. In Vitro Phenolic Release

The release profiles of both RSV and GA bioactive compounds were assessed for later comparison with their release behavior. Both native (compound non-encapsulated) and DPF (compound encapsulated) samples with 1 mg of RSV/GA were assayed. The sample was weighed into a SnakeSkin^®^ (Dialysis Tubing, Thermo Scientific, Waltham, MA, USA) and encapsulated in lozenge shape. The sample was then put into amber flasks containing 25 mL pH 5.5 phosphate-buffered saline (PBS) as a dissolution medium and triplicate experiments were run. Release took place inside an incubator agitator (IKA KS 4000 I control) at 100 rpm at a controlled temperature of 32 °C. Aliquots of 1 mL were withdrawn each time at specific time intervals from 0.03 to 72 h. The amount of compound released through the SnakeSkin^®^ was quantified by HPLC, as previously described for the encapsulation efficiency tests. After in vitro release assays, quantification tests were performed on both RSV–DPF and GA–DPF samples. A known quantity of each DPF was directly dissolved in ethanol in the same volume used in the tests (25 mL). These quantification tests were performed in triplicate for each sample and each solvent.

The transport mechanism associated with the DPF phenolic release was measured using two mathematical models, Higuchi and Korsmeyer–Peppas, according to Equations (6) and (7), respectively [55,64]. These models were fitted for the first 60% of release, regardless of particle geometry.

(6) $$\frac{M_{t}}{M_{\infty }}=kt^{\frac{1}{2}}$$

(7) $$\frac{M_{t}}{M_{\infty }}=kt^{n}$$

The $M_{t}/M_{\infty }$ is defined as the fraction of phenolic compound released at time t, where M_t_ is the phenolic mass released at *t*, and M_∞_ is the initial loading of phenolic in the DPF. The release rate constant (*k*) is obtained from the slope of the representation of $M_{t}/M_{\infty }$ versus *t*^1/2^ according to the Higuchi model. In accordance with Korsmeyer–Peppas, the constant *k* derives from the interception of the representation of natural log ($M_{t}/M_{\infty }$) versus natural log (*t*).

In the Korsmeyer–Peppas equation, the parameter *n* corresponds to the diffusional release exponent and results from the slope of the natural log ($M_{t}/M_{\infty }$) versus natural log (*t*). It will define the type of mechanism associated with the release of RSV/GA. For spherical shapes, the release mechanism may be a contribution of a Fickian diffusion for *n* ≤ 0.43, an anomalous non-Fickian diffusion for 0.43 < *n* < 0.85, or as a Case-II transport for *n* ≥ 0.85 [70]. This model was developed exclusively for the release of an active molecule from a polymeric matrix.

All visuals were represented and treated using OriginPro 2018 software.

## 4. Conclusions

The Supercritical CO_2_-Assisted Spray Drying (SASD) lab-scale apparatus has proven to be effective for producing dry powders of resveratrol (RSV–DPFs) and gallic acid (GA–DPFs) with process yields above 60% and phenolic loadings up to 100%. XRD, while DSC analysis confirmed the amorphous nature of the SASD DPF. According to the ATR-FTIR, bands corresponding to some functional groups of the phenolic compounds were observed in only physical mixtures (PM-RSV and PM-GA), although with very low-intensity peaks. (C_solids) and ethanol percentage (Ethanol (%*v*/*v*)) appeared to be inconclusive for the yield response, while *EE* appeared to be consistent for both RSV and GA–DPFs. Phenolic microparticle loading was higher at level 0s and +1 of both parameters. The volumetric microparticle diameter of both RSV and GA–DPFs was unexpected, with smaller particle sizes for larger solid concentrations in the atomized solution. Finally, only the ethanol percentage affected the results clearly and consistently in the quadratic component for both RSV and GA–DPFs. More homogeneous size distributions were obtained at low and high ethanol levels of this variable in the DPFs with both phenolics, although this does not make the span a decisive parameter when choosing the best combinations since its values were generally below 2. It can be concluded that *EE* is the response with the best combination of factor levels when it comes to DPF selection for antioxidant and in vitro release tests.

In terms of aerodynamic performance, the co-atomization of HPC with the phenolic compounds seems to have improved the powder FPF, from 15–18% with processed HPC without any bioactive compound, to 34–38% for GA–DPFs and 37–47% for RSV–DPFs. These were maximum percentage intervals obtained for DPFs prepared from ethanol percentages of 45 and 70 %*v*/*v* in the atomized solution. However, these results are still not considered ideal for inhalation since a large amount of powder is retained in the induction port in all ACI tests. Nevertheless, SASD has revealed itself to be a suitable technique for preserving the antioxidant activity of thermosensitive compounds using a cellulose derivative as a carrier. The atomization conditions preserved antioxidant activity. Average EC_50_ values of 30.6 µg/mL for GA–DPFs and 149.4 µg/mL for RSV–DPFs coincided with the antioxidant power of the phenolic compound in its pure form.

Regarding the in vitro release in an aqueous medium at pH 5.5 and 32 °C, different behaviors can be concluded for each phenolic. GA exhibited almost complete release of the initial quantity (93.4 ± 0.8%) while the opposite took place for RSV (20 ± 3%) due to its insufficient solubility in aqueous media. Our results show that more research into developing processes for the recovery of natural active compounds is worthwhile since the benefits of a circular bioeconomy are so propitious. Integrating residues and wastes from agri-food industries into new production chains promises an optimized and efficient use of biomass that may very well increase in value over time.

## Figures and Tables

**Table 1:** SASD-processed RSV–DPF results following the 3^2^ full factorial design.

Exp. No.	C_Solids (%*w*/*v*)	Ethanol (%*v*/*v*)	Yield (%)	*EE* (%)	D_v,50_ (µm)	Span
1	2.5	20	49 ± 22	76 ± 1	24 ± 4	1.39 ± 0.06
2	2.5	45	44 ± 6	82 ± 12	22 ± 4	2.2 ± 0.3
3	2.5	70	53 ± 9	91 ± 2	28 ± 4	1.59 ± 0.05
4	5.0	20	52.5 ± 10.5	74 ± 8	19.64 ± 0.04	1.34 ± 0.03
5	5.0	45	55 ± 12	100 ± 2	18 ± 3	2.2 ± 0.4
6	5.0	70	36 ± 21	100 ^1^	19.6 ± 0.6	1.8 ± 0.4
7	7.5	20	56 ± 2	88 ± 6	20 ± 1	1.33 ± 0.04
8	7.5	45	51.5 ± 0.5	100 ^1^	15.6 ± 0.3	1.99 ± 0.02
9	7.5	70	72 ± 2	100 ^1^	15 ± 1	2.8 ± 0.2

^1^ Value considered when the average value of *EE* > 100%.

**Table 2:** Effects component (linear and or quadratic) with *p*-value obtained from ANOVA for each response studied in RSV–DPFs.

	Effect	*p*-Value
Yield (%)	C_solid (%*w*/*v*)	n.s. ^1^
	Ethanol (%*v*/*v*)	n.s. ^1^
*EE* (%)	C_solid (%*w*/*v*) (L)	0.0209
	Ethanol (%*v*/*v*) (L)	0.0042
D_v,50_	C_solid (%*w*/*v*) (L)	0.0079
	Ethanol (%*v*/*v*)	n.s. ^1^
span	C_solid (%*w*/*v*)	n.s. ^1^
	Ethanol (%*v*/*v*) (L)	0.0069
	Ethanol (%*v*/*v*) (Q)	0.0347
	C_solid (L) Ethanol (L)	0.0284

^1^ n.s. means non-significant effect.

**Table 3:** SASD-processed GA–DPF results following the 3^2^ full factorial designs.

Exp. No.	C_Solids (%*w*/*v*)	Ethanol (%*v*/*v*)	Yield (%)	*EE* (%)	D_v,50_ (µm)	Span
1	2.5	20	42.5 ± 0.5	48 ± 4	22 ± 2	1.37 ± 0.08
2	2.5	45	21 ± 5	100 ^1^	26 ± 8	2.0 ± 0.8
3	2.5	70	58.5 ± 3.5	88 ± 11	30 ± 1	1.8 ± 0.1
4	5.0	20	55 ± 3	65 ± 7	22 ± 4	1.5 ± 0.2
5	5.0	45	41 ± 15	94 ± 6	18 ± 4	2.2 ± 0.8
6	5.0	70	58 ± 2	100 ^1^	23 ± 2	2.0 ± 0.2
7	7.5	20	50.5 ± 2.5	78 ± 7	21 ± 1	1.34 ± 0.02
8	7.5	45	58 ± 5	100 ^1^	14 ± 2	2.3 ± 0.2
9	7.5	70	61 ± 2	96 ± 3	16 ± 2	1.9 ± 0.2

^1^ Value considered when the average value of *EE* > 100%.

**Table 4:** Effects component (linear and or quadratic) with ANOVA *p*-value for each GA–DPFs.

	Effect	*p*-Value
Yield (%)	C_solid (%*w*/*v*) (L)	0.0092
	Ethanol (%*v*/*v*) (Q)	0.0075
	C_solid (L) Ethanol (Q)	0.0123
*EE* (%)	C_solid (%*w*/*v*) (L)	0.0178
	Ethanol (%*v*/*v*) (L)	0.0002
	Ethanol (%*v*/*v*) (Q)	0.0070
D_v,50_	C_solid (%*w*/*v*) (L)	0.0162
	Ethanol (%*v*/*v*)	n.s. ^1^
span	C_solid (%*w*/*v*)	n.s. ^1^
	Ethanol (%*v*/*v*)	n.s. ^1^

^1^ n.s. means non-significant effect.

**Table 5:** Specific surface area (m^2^/g) of both raw phenolics and respective RSV and GA–DPFs.

Sample	BET Specific Surface Area (m^2^/g)
HPC 2.5 %*w*/*v*	1.95
HPC 5.0 %*w*/*v*	2.62
HPC 7.5 %*w*/*v*	2.08
raw RSV	2.19
raw GA	1.25
RSV–DPF	4.20
GA–DPF	2.04

**Table 6:** EC_50_ values of unprocessed and SASD-processed RSV and GA.

EC_50_					
Sample	(µM)	(µg/mL)	Sample	(µM)	(µg/mL)
raw RSV	528.99	120.74	raw GA	125.55	21.36
DPF RSV/5.0/45	807.96	184.41	DPF GA/5.0/45	163.40	37.29
DPF RSV/7.5/20	672.43	153.47	DPF GA/7.5/20	122.16	27.88
DPF RSV/7.5/70	483.51	110.36	DPF GA/7.5/70	117.28	26.77

**Table 7:** Kinetic constants obtained through the fitting of the RSV and GA release profiles (pH 5.5 PBS and at 32 °C) using the Higuchi and Korsmeyer–Peppas mathematical models.

	Higuchi		Korsmeyer–Peppas		
	*k* (min^−0.5^)	R^2^	*k* (min^−n^)	*n*	R^2^
RSV–DPF	0.0055 ^†^	0.9975 ^†^	0.0001 ^‡^	1.1518 ^‡^	0.9932 ^‡^
GA–DPF	0.0507	0.9950	0.0051	0.9870	0.9948

^†^ Fitted up to 16.4% of RSV release; ^‡^ Fitted up to 21.2% of RSV release.

## Data Availability

All data generated or analyzed during the present study are included in this published article.

## Supplementary Materials

The following supporting information can be downloaded at: https://www.mdpi.com/article/10.3390/molecules27062001/s1, Figure S1: Schematic representation of the 32 full factorial DoE.; Figure S2: Morphologi G3 images of the DPFs microparticles with 2.5, 5.0 and 7.5 %*w*/*v* of HPC at different image magnifications: 10,000×, 20,000× and 50,000×; Figure S3: XRPD diffraction spectra of raw HPC powder and DPFs with 2.5, 5.0 and 7.5 %*w*/*v* HPC.; Figure S4: SEM images of raw GA and raw RSV at 500× and 1000× magnifications; Figure S5: Isotherm plots for (a) HPC/RSV and (b) HPC/GA microparticles obtained by SASD.; Figure S6: In vitro phenolic release profiles in pH 5.5 PBS at 32 °C for 24 h from (a) raw RSV and DPF RSV/5.0/45 samples and (b) raw GA and DPF GA/5.0/45 samples, expressed in µg of phenolic per mL of solution.; Table S1: Constant SASD parameters.; Table S2: Process yield and physicochemical characteristics of the SASD HPC powders.; Table S3: Parameter level values used in 32 full factorial design.; Table S4: Standard experiment order matrix.; Table S5: DPPH radical scavenging (%) of RSV tests.; Table S6: DPPH radical scavenging (%) of GA tests.; Table S7: Input and released amount of RSV and GA from the in vitro release tests.

## Sample Availability

Not available.
