# Supplementary material for: Dry Dosage Forms of Add-Value Bioactive Phenolic Compounds by Supercritical CO2-Assisted Spray-Drying
Source: Molecules. 2022 Mar 20;27(6):2001. doi: 10.3390/molecules27062001 (PMC8950927; doi:10.3390/molecules27062001)
Supplement: Supplementary file 1 [file molecules-27-02001-s001.zip › molecules-1625562-supplementary.pdf]

Supplementary material

Table S1–Constant SASD parameters.

F_{CO2} (mL/min)	F_{Feed} (mL/min)	T_{in} (ºC)	T_{SM} (ºC)	Th_{CO2} (ºC)	Tc_{CO2} (ºC)	P_{SM} (MPa)	d_{nozzle} (μm)
25	3.5	100	80	80	–20	10	150

F_{CO2}: CO_2 flow rate; F_{Feed}: liquid feed solution flow rate; T_{in}: inlet drying gas temperature; T_{SM}: saturaturation temperature; Th_{CO2}: CO_2 oil bath temperature; Tc_{CO2}: CO_2 cooling bath temperature; P_{sat}: saturation pressure

Table S2–Process yield and physicochemical characteristics of the SASD HPC powders .

**HPC (%w/v)**	**Yield, η (%)**	**$D_{v,50}$ (μm)**	**span**	**Moisture Content (%H_2O/g_{powder})**	**a_{BET} (m^2/g)**	**SEM (shape/ surface)**	**XRPD (solid-state)**
2.5	34	20.24	1.39	n.a.*	1.95	n.a.	Amorphous
5.0	66	19.95	1.44	4.3 ± 0.6	2.62	n.a.	Amorphous
7.5	49	18.26	1.52	4.6 ± 0.4	2.08	Irregular/ smooth	Amorphous
Raw	n.a.	n.a.	n.a.	2.5 ± 0.4	0.80	n.a.	Amorphous

* Test not performed due to the insufficient powder amount required for the characterization; n.a. = not available.

Table S3–Parameter level values used in 3^2 full factorial design.

		Level of factors used in the formulation		
Factor		–1	0	+1
A	C_solids (%w/v)	2.5	5.0	7.5
B	Ethanol (%v/v)	20	45	70

C_solids: solid contents

Levels: low (–1), medium (0) and high (+1).

Table S4–Standard experiment order matrix.

Exp. No.	A	B
1	–1	–1
2	–1	0
3	–1	+1
4	0	–1
5	0	0
6	0	+1
7	+1	–1
8	+1	0
9	+1	+1

Table S5–DPPH radical scavenging (%) of RSV tests.

C_{RSV} (μM)	raw RSV	RSV / 5.0 / 45	RSV / 7.5 / 20	RSV / 7.5 / 70
**100**	**10.75 ± 0.08**	**7.30 ± 0.32**	**11.54 ± 0.34**	**9.79 ± 0.31**
**400**	**28.40 ± 0.75**	**15.22 ± 0.75**	**19.32 ± 1.33**	**22.33 ± 0.57**
**800**	**43.01 ± 0.73**	**24.54 ± 0.67**	**33.60 ± 3.36**	**34.90 ± 0.34**
**1200**	**48.57 ± 5.63**	**38.63 ± 4.29**	**48.27 ± 0.33**	**43.86 ± 0.28**
**1500**	**53.75 ± 5.74**	**37.25 ± 7.21**	**52.26 ± 1.01**	**47.60 ± 0.35**
**1800**	**66.22 ± 2.50**	**46.87 ± 0.46**	**55.45 ± 0.70**	**43.49 ± 2.81**
**2100**	**62.24 ± 1.32**	**49.64 ± 0.63**	**58.09 ± 0.50**	**49.92 ± 0.74**

Table S6–DPPH radical scavenging (%) of GA tests.

C_{RSV} (μM)	raw GA	GA / 5.0 / 45	GA / 7.5 / 20	GA / 7.5 / 70
**50**	**11.31 ± 7.18**	**8.47 ± 1.54**	**15.18 ± 1.13**	**9.48 ± 4.58**
**120**	**31.50 ± 1.04**	**24.44 ± 7.98**	**41.67 ± 2.72**	**43.72 ± 1.83**
**190**	**60.67 ± 1.15**	**49.96 ± 8.20**	**75.95 ± 3.64**	**76.82 ± 4.25**
**260**	**79.00 ± 5.48**	**59.51 ± 13.75**	**82.97 ± 2.42**	**82.92 ± 1.25**
**330**	**88.54 ± 0.15**	**59.69 ± 14.34**	**85.46 ± 0.16**	**81.12 ± 3.55**
**400**	**89.17 ± 0.46**	**61.58 ± 10.72**	**75.47 ± 1.18**	**83.86 ± 0.52**
**470**	**90.00 ± 0.12**	**80.53 ± 3.46**	**76.10 ± 1.01**	**84.79 ± 1.25**

Table S7–Input and released amount of RSV and GA from the *in vitro* release tests.

Sample	C_{input} (μg/mL)	$\mu g_{\text{phenolic input}}$/ 100mg DPF	$C_{máx}$ (μg/mL)	$\mu g_{\text{phenolic rel.}}$/ 100mg DPF	$\%_{released}$
Raw RSV	40.9 ± 0.2	–	1.4 ± 0.4	–	3 ± 1
DPF RSV/5.0/45	40.714 ± 0.008	1017	8 ± 1	201 ± 32	20 ± 3
Raw GA	41.7 ± 0.7	–	37 ± 2	–	92 ± 3
DPF GA/5.0/45	40.354 ± 0.006	877	37.2 ± 0.2	818 ± 6	93.4 ± 0.8

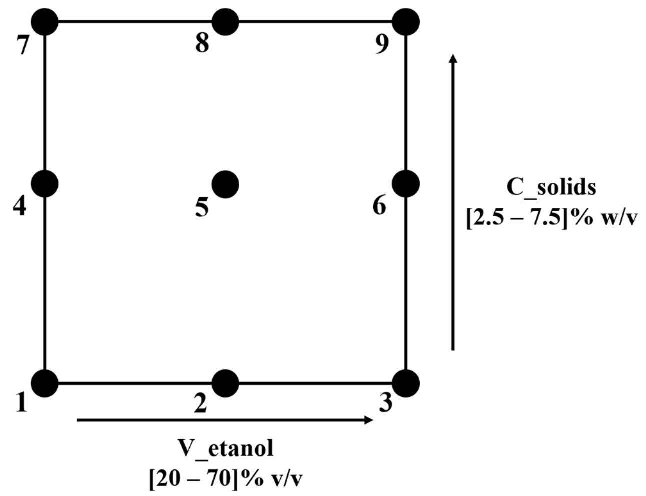

Figure S1–Schematic representation of the 3^2 full factorial DoE.

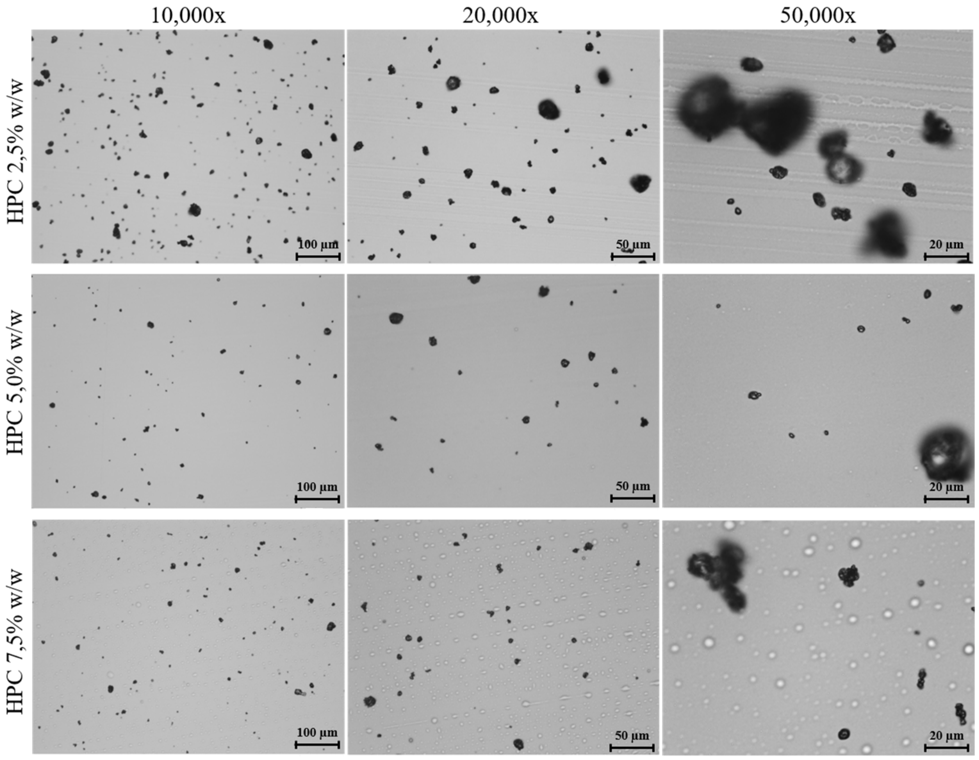

Figure S2–Morphologi G3 images of the DPFs microparticles with 2.5, 5.0 and 7.5 %w/v of HPC at different image magnifications: 10,000×, 20,000× and 50,000×.

·

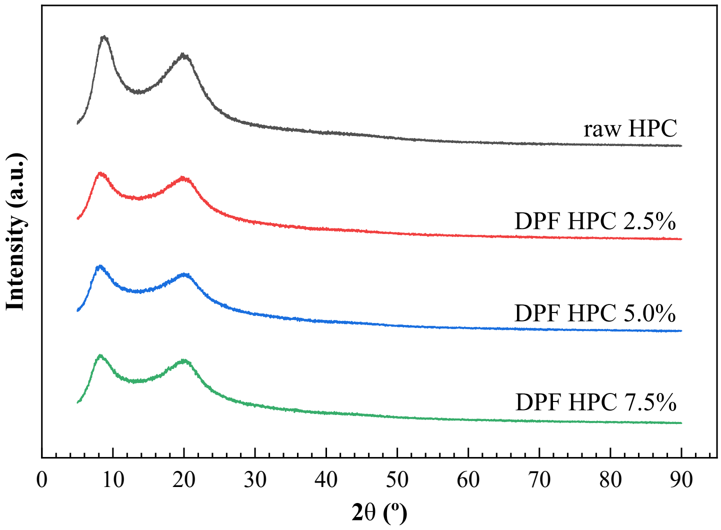

Figure S3–XRPD diffraction spectra of raw HPC powder and DPFs with 2.5, 5.0 and 7.5 %w/v HPC.

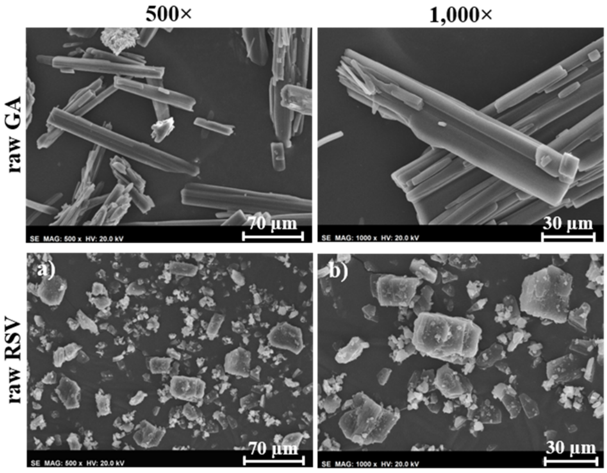

Figure S4–SEM images of raw GA and raw RSV at 500× and 1,000× magnifications.

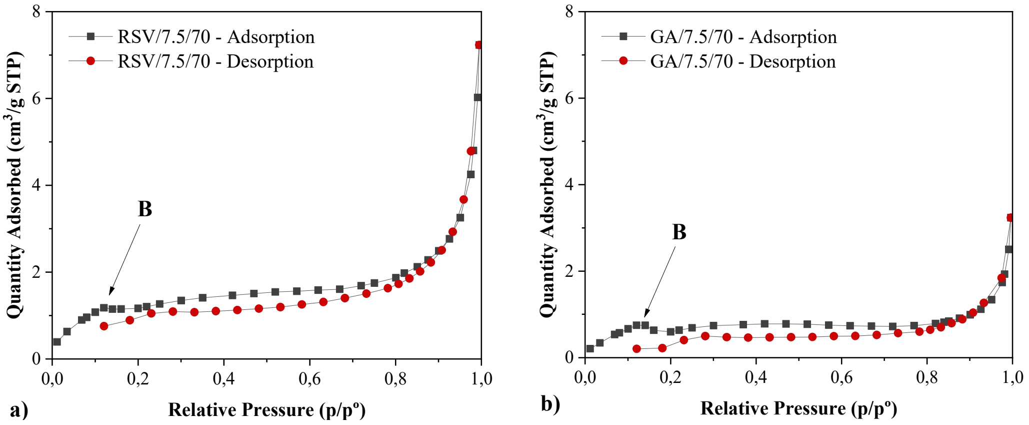

Figure S5–Isotherm plots for a) HPC/RSV and b) HPC/GA microparticles obtained by SASD.

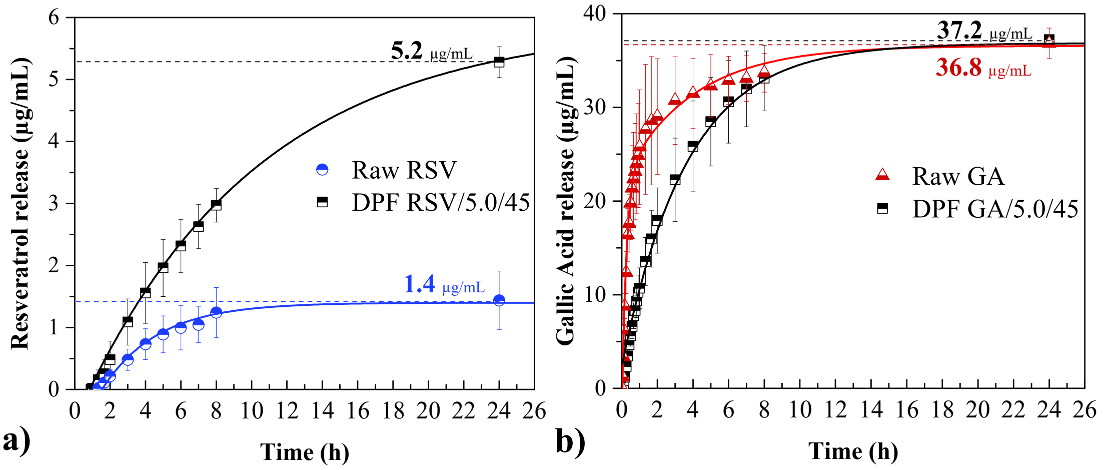

Figure S6–In vitro phenolic release profiles in pH 5.5 PBS at 32 ºC for 24 hours from a) raw RSV and DPF RSV/5.0/45 samples and b) raw GA and DPF GA/5.0/45 samples, expressed in µg of phenolic per mL of solution.
